# The cytoskeletal crosslinking protein MACF1 is dispensable for thrombus formation and hemostasis

**DOI:** 10.1038/s41598-019-44183-6

**Published:** 2019-05-22

**Authors:** Yvonne Schurr, Markus Spindler, Hendrikje Kurz, Markus Bender

**Affiliations:** Institute of Experimental Biomedicine – Chair I, University Hospital and Rudolf Virchow Center, Würzburg, Germany

**Keywords:** Microtubules, Actin

## Abstract

Coordinated reorganization of cytoskeletal structures is critical for key aspects of platelet physiology. While several studies have addressed the role of microtubules and filamentous actin in platelet production and function, the significance of their crosstalk in these processes has been poorly investigated. The microtubule-actin cross-linking factor 1 (MACF1; synonym: Actin cross-linking factor 7, ACF7) is a member of the spectraplakin family, and one of the few proteins expressed in platelets, which possess actin and microtubule binding domains thereby facilitating actin-microtubule interaction and regulation. We used megakaryocyte- and platelet-specific Macf1 knockout (*Macf1*^*fl/fl*^*, Pf4-Cre*) mice to study the role of MACF1 in platelet production and function. MACF1 deficient mice displayed comparable platelet counts to control mice. Analysis of the platelet cytoskeletal ultrastructure revealed a normal marginal band and actin network. Platelet spreading on fibrinogen was slightly delayed but platelet activation and clot traction was unaffected. *Ex vivo* thrombus formation and mouse tail bleeding responses were similar between control and mutant mice. These results suggest that MACF1 is dispensable for thrombopoiesis, platelet activation, thrombus formation and the hemostatic function in mice.

## Introduction

Platelets are anucleated cell fragments derived from megakaryocytes (MKs) in the bone marrow. During maturation, MKs undergo cell growth, become polyploid and develop the demarcation membrane system, which forms the plasma membrane for future platelets. MKs extend long protrusions, so-called proplatelets, into sinusoidal blood vessels where platelets are finally shed into the blood stream by shear forces^1,2^. Cytoskeletal dynamics have been shown to be critical in the terminal stages of platelet formation. While microtubule sliding enables proplatelet elongation^3,4^, actin-dependent mechanisms are important for maturation of the demarcation membrane system, the initiation of proplatelet formation and branching of proplatelet shafts^5–7^. Consequently, deficiency of actin- or microtubule-modulating proteins in MKs has been frequently associated with impaired platelet production^8^. Furthermore, mouse models particularly for actin binding and regulatory proteins, such as gelsolin, profilin 1, Arp2/3 and twinfilin, provided evidence for a role of the cytoskeleton in platelet morphology, shape change and activation^9–12^.

The cytoskeleton, however, is no longer considered as a collection of individual proteins but rather as a system in which the components work with and co-regulate each other to exert their cellular function^13^. Spectraplakins are evolutionary conserved proteins and unusual in their capacity to simultaneously bind and regulate microtubules and filamentous actin (F-actin)^14^. The spektraplakin family only consists of two mammalian members, the Microtubule-actin cross-linking factor 1 (MACF1; also known as ACF7; actin cross-linking factor 7) and Dystonin (Dst, also known as bullous pemphigoid antigen 1, Bpag1)^15^. Spektraplakins (300–800 kDa) carry two calponin-homology domains that facilitate the interaction with F-actin, a plakin domain, a spectrin-repeat domain, an EF hand motive, and a GAR domain, which is responsible for binding to microtubules^16^. Full length MACF1 is ubiquitously expressed, whereas Dystonin is mainly present in the brain^15,17^. In endodermal cells, MACF1 was described as an essential integrator of actin-dependent microtubule dynamics^18^, and a study in mouse keratinocytes revealed that MACF1 deficiency compromises the targeting of microtubules along F-actin to focal adhesions^19^. Further, Macf1 knockdown in neuronal culture systems demonstrated that MACF1 is essential for axon extension and the organization of neuronal microtubules, a function dependent on both the microtubule and F-actin-binding domains^20^. These data demonstrate that the crosstalk between cytoskeletal components is essential for the orchestration of basic cellular functions.

This crosstalk, however, has only been poorly studied in platelet biology, and the role of MACF1 in MKs and platelets is unknown. Therefore, we used MK- and platelet-specific knockout mice to investigate the function of MACF1 in platelet formation, *in vitro* platelet activation as well as aggregation.

## Results

### MACF1 deficiency does not affect platelet count, size or other blood parameters

**Figure 1:**
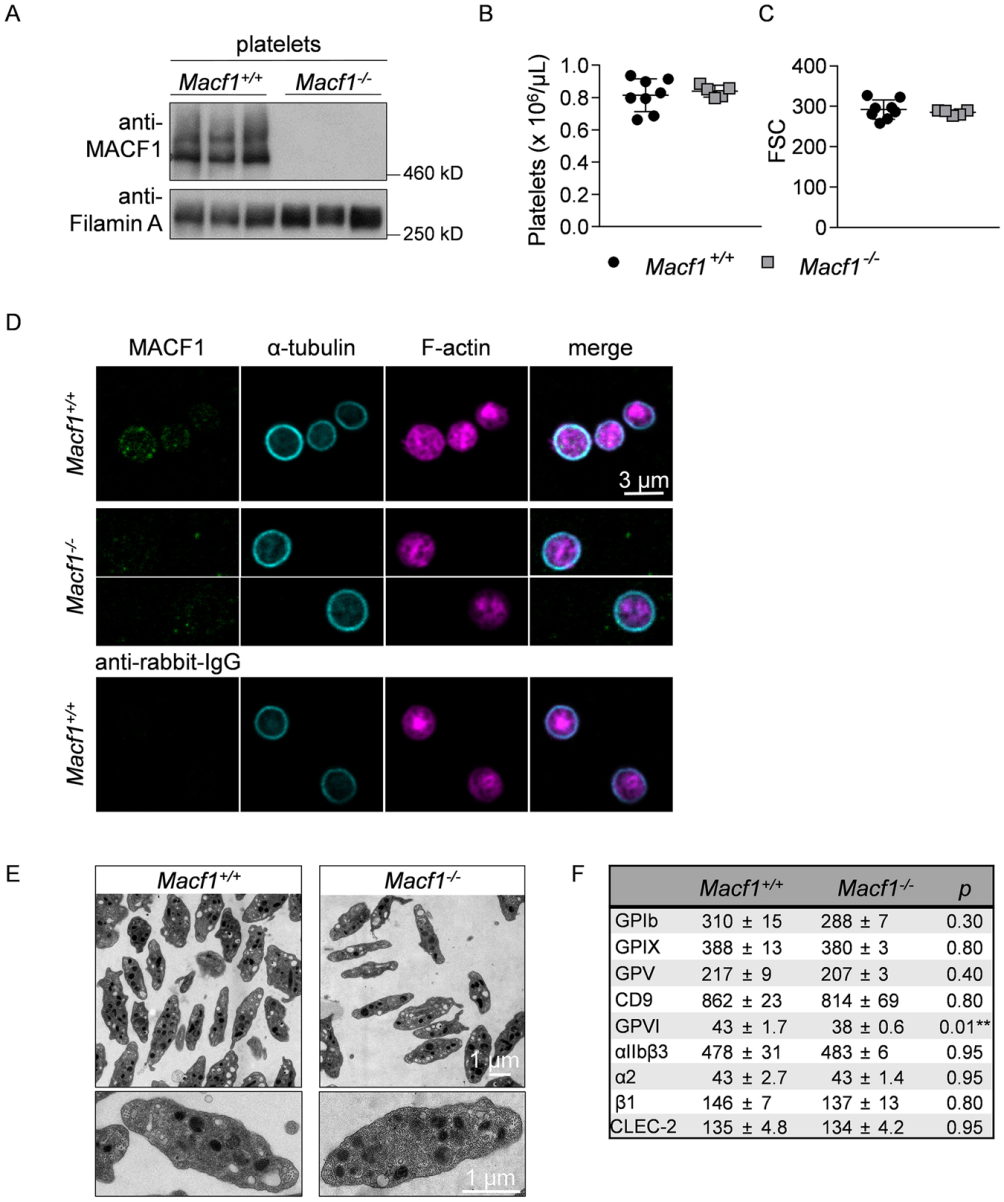
MACF1 deficiency has no effect on platelet count and size. (**A**) Western blot analysis of MACF1 expression in platelets. Loading control: Filamin A. **(B)** Platelet count per μL and **(C)** platelet size given as mean forward scatter (FSC) were determined via flow cytometry. **(D)** Immunostaining of resting platelets on Poly-L-lysine. Green: MACF1/ACF7, cyan: α-tubulin, purple: F-actin. Scale bar: 3 µm. **(E)** Representative transmission electron micrographs (TEM) from *Macf1*^+/+^ and *Macf1*^−/−^ platelets. **(F)** Determination of glycoprotein expression by flow cytometry. Diluted whole blood was incubated with FITC-labelled antibodies and measured as mean fluorescence intensity (MFI); (n = 3); **P < 0.01. **(A,D,E)** Representative of at least 3 versus 3 samples.

Due to embryonic lethality of constitutive MACF1 deficient mice on day 11.5 (E11.5)^21^, we capitalized on MK-/platelet-specific knockout mice (*Macf1*^*fl/fl, Pf4-Cre*^ further referred to as *Macf1*^−/−^) and the respective littermate controls (*Macf1*^*fl/fl*^ further referred to as *Macf1*^*+/+*^). *Macf1*^−/−^ mice were viable, fertile, and born at normal Mendelian ratio (data not shown). Absence of MACF1 in mutant MKs and platelets was confirmed by Western blot analysis with three different antibodies, whereas two specific MACF1 bands, conferring to the isoforms Macf1a1-3 (500–620 kDa) and Macf1b (800 kDa), were detectable in lysates of control samples (Fig. 1A and Suppl. Fig. 1A)^15,22^. Count of platelets and other blood cells was comparable between *Macf1*^*+/+*^ and *Macf1*^−/−^ mice as determined by flow cytometry and a hematology analyzer (Fig. 1B,C and Suppl. Fig. 1B). Platelet size, shape and ultrastructure were unaltered in *Macf1*^−/−^ mice, as confirmed by flow cytometry (Fig. 1C) and transmission electron microscopy (TEM) (Fig. 1E), respectively. We investigated the localization of MACF1 in resting platelets on Poly-L-lysine. Immunostainings^19^ revealed a ring-shaped expression of MACF1 close to the membrane and only a weak localization in the platelet cytoplasm (Fig. 1D). This result indicates that MACF1 might crosslink microtubules and F-actin at the platelet membrane. Analysis of platelet receptors by flow cytometry revealed only a minor decrease in GPVI expression on mutant platelets (Fig. 1F). We further investigated the MKs in the bone marrow and spleen of MACF1 deficient mice. Localization, number as well as ploidy of MKs were unaltered in the bone marrow (Suppl. Fig. 2A–C). The structure and size of the spleen appeared normal, and the number of MKs was comparable between control and mutant mice (Suppl. Fig. 2D–F). Additionally, we observed that fetal liver cell-derived MKs of mutant mice formed proplatelets to the same extent as control MKs (Suppl. Fig. 3A,B). These data suggest that MACF1 is dispensable for platelet production.

### Unaltered inside-out activation and aggregation of *Macf1*^−/−^ platelets

**Figure 2:**
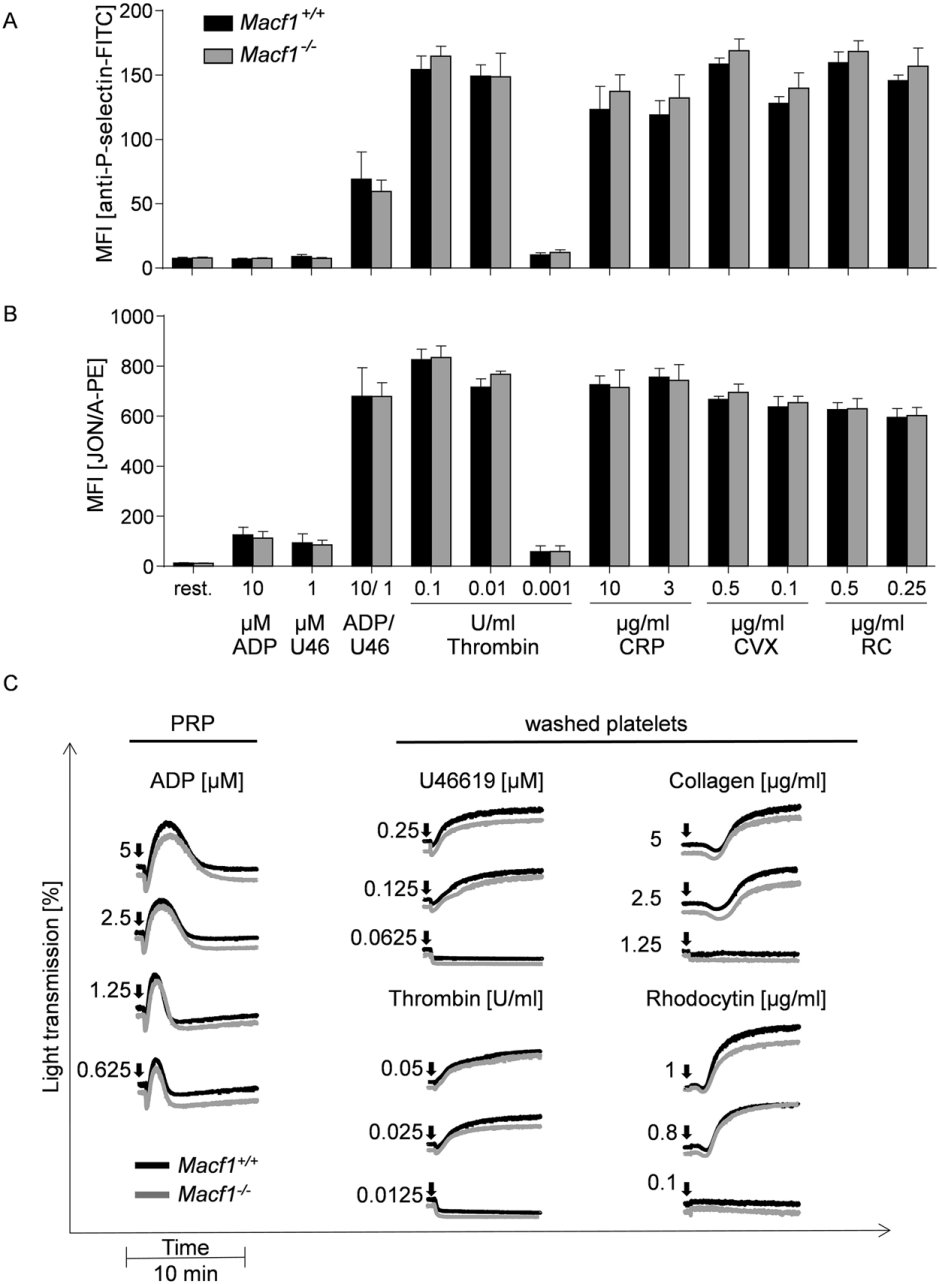
Unaltered P-selectin exposure, αIIbβ3 integrin activation and aggregation responses to GPCR, GPVI and CLEC-2-dependent agonists of *Macf1*^−/−^ platelets. **(A)** Degranulation dependent P-selectin exposure using a FITC-labelled anti-P-selectin antibody and **(B)** inside-out activation of αIIbβ3 integrin using the JON/A-PE antibody (n = 6, representative for four independent experiments) in response to agonists ADP, U46619 (thromboxane analogue), Thrombin, collagen-related peptide (CRP), convulxin (CVX) and Rhodocytin (RC) were determined by flow cytrometry. **(C)** Aggregation studies: Platelet rich plasma (PRP) or washed platelets from *Macf1*^+/+^and *Macf1*^−/−^ mice were activated with the indicated agonist concentrations (n = 3–8).

Next, we studied the role of MACF1 in platelet function. Therefore, surface exposure of P-selectin and activation of the platelet integrin αIIbβ3 was analyzed after the incubation with different agonists. Comparable results for control and *Macf1*^−/−^ platelets were obtained after stimulation of G-protein coupled receptors with ADP, the thromboxane A2 analogue U46619, a combination of both, or thrombin. Similarly, MACF1 deficient platelets exhibited comparable degree of activation upon stimulation of the GPVI-ITAM (immunoreceptor tyrosine-based activation motif) pathway with collagen-related peptide (CRP) or convulxin, and of the hemITAM receptor, CLEC-2 (C-type lectin-like receptor 2), with rhodocytin (Fig. 2A,B). To clarify the role of MACF1 for platelet shape change and aggregation, *in vitro* aggregation studies were performed. All agonists induced a comparable activation-dependent change from discoid to spherical shape of control and *Macf1*^−/−^ platelets, which can be seen in aggregometry as a short decrease in light transmission following the addition of agonists. Further, *Macf1*^−/−^ platelets showed a normal onset and degree of aggregation (Fig. 2C).

### Lack of MACF1 has no impact on the cytoskeletal structure and reorganization in platelets

**Figure 3:**
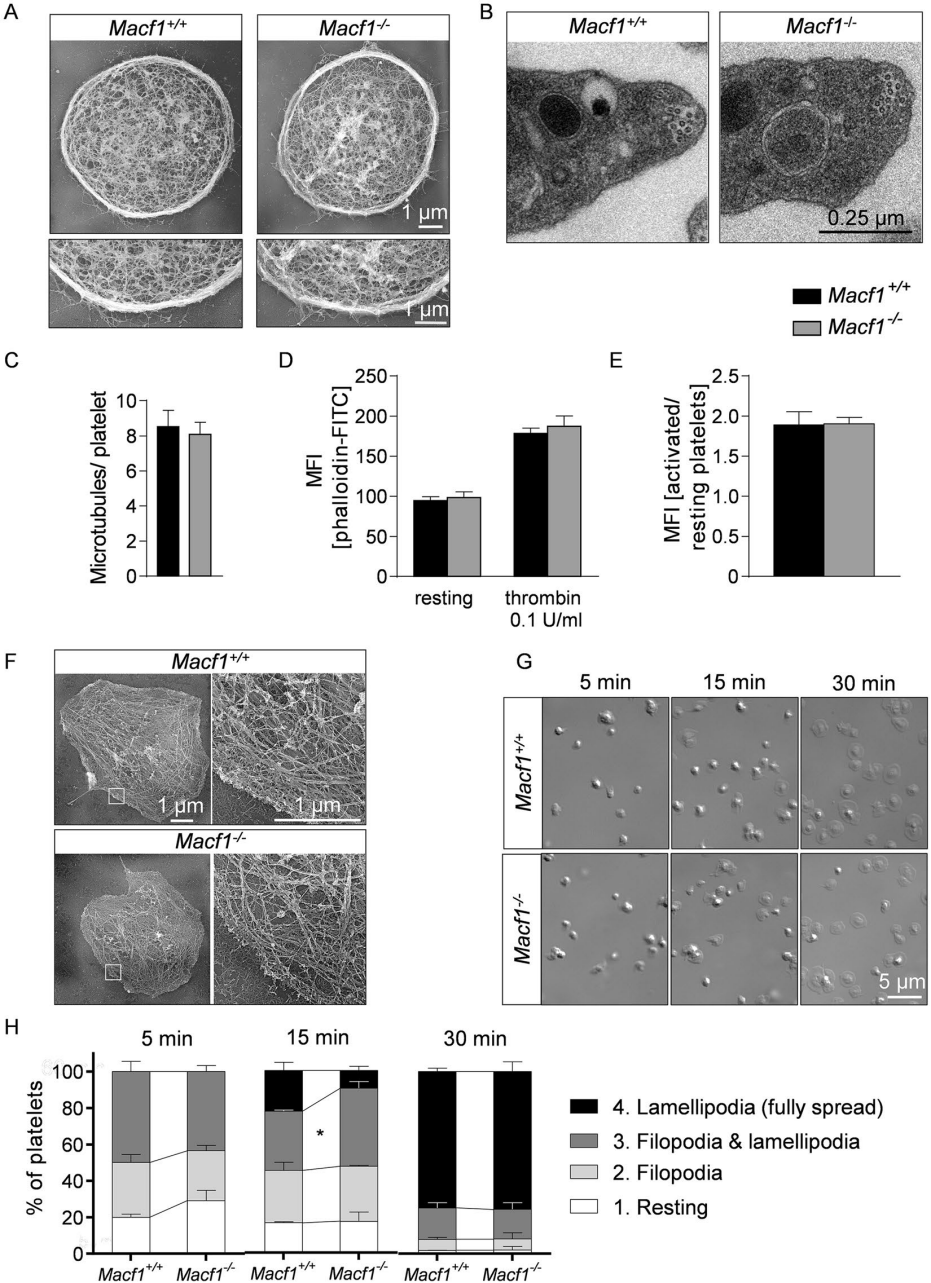
Lack of MACF1 does not affect microtubule and actin arrangement. **(A)** Images of the platelet cytoskeleton ultrastructure on poly-L-lysine (n = 2). **(B)** TEM images of resting platelets (n = 4) and **(C)** evaluation of microtubule number. **(D)** Resting or thrombin-activated platelets were fixed, permeabilized, stained with phalloidin-FITC, and analyzed by flow cytometry. **(E)** The ratio of polymerized actin in activated vs. resting platelets was determined (n = 3, representative for two independent experiments). **(F)** Representative transmission electron micrographs of the cytoskeleton ultrastructure of platelets spread on fibrinogen in the presence of 0.01 U/ml thrombin (n = 2). **(G)** Representative images and **(H)** statistical analysis of the different spreading phases of fixed *Macf1*^+/+^ and *Macf1*^−/−^ platelets on fibrinogen at different time points (n = 3). *P < 0.05.

In various cell types, MACF1 has been shown to be important for crosslinking microtubules and F-actin and thereby controlling cytoskeletal rearrangement^15^. This prompted us to investigate whether MACF1 is also a crucial regulator of cytoskeletal dynamics in platelets. We first analyzed the cytoskeletal organization of resting *Macf1*^−/−^ platelets. TEM analysis revealed a normal actin scaffold and the characteristic ring structure organized by microtubules, designated as the marginal band (Fig. 3A). Next, we quantified the number of microtubule coils per platelet. MACF1 deficient platelets had a similar number of microtubules compared to controls (Fig. 3B,C). The cold resistance of microtubules was analyzed by incubating platelets at different temperatures to induce dis- or reassembly of microtubules. Fixation and staining for α-tubulin of the platelets revealed comparable dis- and reassembly of microtubules (Suppl. Fig. 4A). Further, platelet F-actin assembly after activation with thrombin was normal in *Macf1*^−/−^ platelets (Fig. 3D,E). To investigate the organization and rearrangement of the cytoskeleton in *Macf1*^−/−^ platelets, we performed spreading experiments on a fibrinogen-coated surface. Mutant platelets were able to rearrange the cytoskeleton and form filopodia and lamellipodia as revealed by TEM analysis after removing the platelet membrane (Fig. 3F time point 15 minutes) and by differential interference contrast imaging (Fig. 3G). We only observed a significantly delayed transition of mutant platelets forming filopodia and lamellipodia to platelets forming lamellipodia only (fully spread) at the 15 min time point. After 30 min the number of fully spread platelets was comparable (Fig. 3H). We also assessed whether MACF1 deficiency might impair the cytoskeletal reorganization when platelets are treated with cytoskeletal-modifying toxins, such as colchicine, latrunculin A or paclitaxel. However, we could not observe any effect of MACF1 deficiency on platelet spreading in the presence of these toxins (Suppl. Fig. 4B).

### MACF1 is not important for thrombus formation, clot retraction and hemostasis

**Figure 4:**
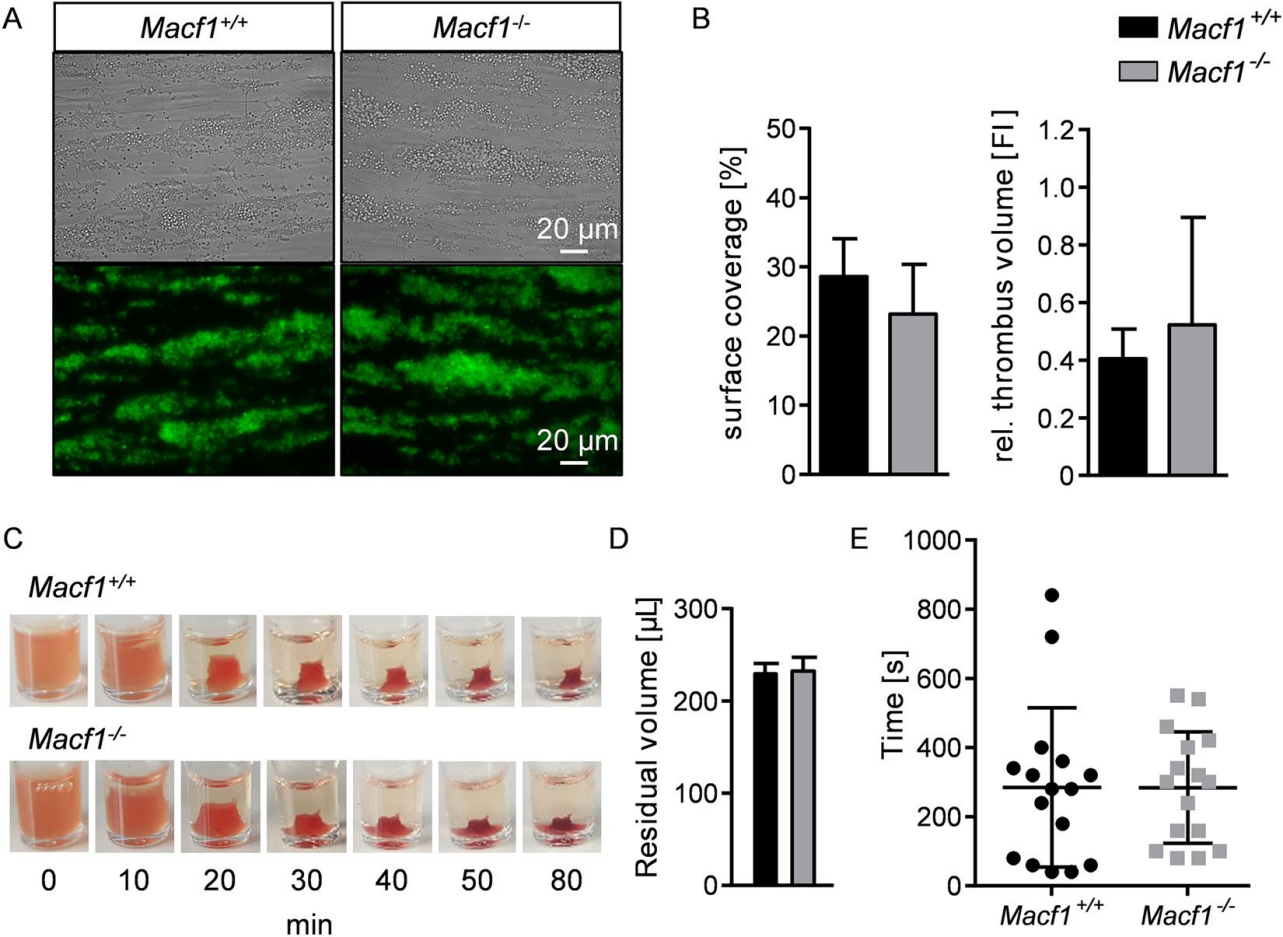
MACF1 is not important for thrombus formation, clot retraction and hemostasis. **(A)** Thrombus formation was assessed on collagen at a wall shear rate of 1,000 s^−1^. Shown are representative bright field (upper panel) and fluorescence (lower panel) pictures of platelets stained with a Dylight-488 anti-GPIX antibody. (n = 6, representative for two independent experiments) **(B)** Surface coverage and relative thrombus volume was quantified according to fluorescence distribution and fluorescence intensity, respectively. **(C)** Clot formation was observed over time and **(D)** residual serum volume was measured. (n = at least 3, representative for three independent experiments) **(E)** Determination of tail bleeding time performed on filter paper of *Macf1*^+/+^ and *Macf1*^−/−^ mice.

Under physiological conditions, platelet adhesion and aggregation occur in the peripheral blood stream where shear forces strongly influence platelet function. To test the role of MACF1 in thrombus formation under flow, we studied platelet adhesion to collagen in a whole-blood perfusion assay at shear rates of 1,000 s^−1^. Control and mutant platelets rapidly adhered to collagen and consistently formed stable 3-dimensional thrombi (Fig. 4A). Surface coverage and thrombus volume were comparable between blood from *Macf1*^*+/+*^ and *Macf1*^−/−^ mice indicating that MACF1 is dispensable for thrombus formation (Fig. 4B). To further assess integrin outside-in signalling, which is an important mechanism to stabilize thrombi, we performed a clot retraction assay and found no differences in the kinetics of clot retraction between PRP from both mouse strains (Fig. 4C,D). In addition, comparable bleeding times were measured for control and mutant mice, indicating that MACF1 is not required to maintain the hemostatic function (Fig. 4E). Taken together, these data demonstrate that MACF1 is dispensable for thrombus formation and hemostasis.

## Discussion

In the present study, MK-/platelet-specific knockout mice were analyzed to assess the role of the cytoskeletal crosslinker protein MACF1 for platelet production and function. MACF1 has been described to be an important regulator of microtubules and F-actin in various cell types, such as endodermal cells^18^, keratinocytes^19^ and neurons^20^. However, we did not detect alterations in platelet production, platelet activation, clot retraction, thrombus formation and hemostatic function in MACF1 deficient mice. This result is surprising because platelets undergo intensive shape change, activate integrins, release granules and transduce mechanical forces to fulfill their function, and these processes strongly depend on cytoskeletal rearrangement. Overall, we only found a subtle difference in spreading kinetics at the 15 min time point. Thus, our experimental findings did not reveal an important role of MACF1 in platelet physiology.

However, we detected two specific MACF1 protein bands, conferring to the known Macf1a1-3 (500–620 kDa) and Macf1b (800 kDa) isoforms^15^, which were missing in lysates of MACF1 deficient platelets and MKs, demonstrating the loss of the protein in those cells (Fig. 1A and Suppl. Fig. 1A). We can exclude a compensatory mechanism by the other spektraplakin family member Dystonin, since we could not detect the isoforms, Dystonin a and Dystonin b, by RT-PCR in control or mutant platelets (data not shown). This finding is supported by proteomics studies on human and mouse platelets, which found MACF1 but not Dystonin expressed in platelets^23,24^.

Over the last few years, different ways of crosstalk between the actin and microtubule cytoskeleton have been discovered, ranging from direct physical crosslinks to more indirect mechanisms, such as shared regulators of dynamical properties^13^. Other physical crosslinker proteins, besides spectraplakins, are the growth arrest-specific 2-like (GAS2-like) proteins. Members of the GAS2 family resemble spectraplakins as they possess both actin- and microtubule-binding regions, but they lack the plakin domains^25^. Interestingly, Preciado López *et al*. engineered a minimal version of MACF1, the actin-binding, microtubule plus-end tracking protein, called TipAct, where the plakin domain and spectrin-repeat rod was replaced by a short CC-linker (coiled-coil linker of cortexillin I, which induces parallel dimerization)^26^. This short version was sufficient to crosslink and to enable a mechanical feedback between actin and microtubule organization, implying that TipAct contains the key functional domains of spectraplakins. Thus, the GAS2 protein family might compensate for the lack of MACF1 in the knockout platelets. This family consists of the four members GAS2, GAS2-like 1 (Gas2L1), GAS2-like 2 and GAS2-like 3, of which only Gas2L1 seems to be expressed in platelets (protein copy number: 3074 copies of Gas2L1 and 1632 copies of MACF1 in mouse platelets^23^; 1600 copies of Gas2L1 and 1300 copies of MACF1 in human platelets^24^). We determined whether Gas2L1 is up- or downregulated in knockout platelets but could not detect altered expression (Suppl. Fig. 5). Formins are another group of cytoskeletal crosstalk regulators with DAAM1, mDia1 (DIAPH1) and FHOD1 as the major proteins expressed in platelets^27^. Studies on mDIA knockout mice revealed that the deficiency does not alter platelet activation, spreading and aggregation, presumably due to functional redundancy with other formin proteins^27,28^. However, a role of this protein in platelet formation was demonstrated by DIAPH1 knockdown in cultured human MKs^29^ and by the description of two unrelated pedigrees with a DIAPH1 R1213* variant^30^. Therefore, we tested the expression of DAAM1, mDia1 and the direct binding protein profilin 1, which was recently described by us to be an important regulator of actin and microtubule structure and dynamics in platelets. However, we could also not find a compensatory up- or downregulation of these proteins in mutant platelets (Suppl. Fig. 5). Nevertheless, it is intriguing to speculate that functional redundancy among different proteins mediating the cytoskeletal crosstalk may exist. But this requires further investigation.

## Material and Methods

### Animals

*Macf1*^*fl/fl*^ mice were intercrossed with mice carrying the Cre-recombinase under the platelet factor 4 (*Pf4*) promoter^31^ to generate platelet- and MK-specific Macf1-knockout (further referred to as *Macf1*^−/−^) mice. *Macf1*^*fl/fl*^ mice were obtained from the Jackson Laboratory and initially generated and described by Elaine Fuchs^19^. Male and female mice were analyzed (6–16 week old). Genotyping of mice was performed by PCR with 5′AAAGAAACGGAAATACTGGCC3′ and 5′GCAGCTTAATTCTGCCAAATTC3′ primers for floxed *Macf1* and with 5′CTCTGACAGATGCCAGGACAQ3′ and 5′TCTCTGCCCAGAGTCATCCT3′ primers for Pf4-Cre. All animal studies were approved by the district government of Lower Franconia (Bezirksregierung Unterfranken; AZ: 2-130 to M.B.) and performed in accordance with relevant guidelines and regulations.

### Platelet preparation

Platelet-rich plasma (PRP) was obtained by centrifugation at 80 *g* for 5 minutes at room temperature (RT). For preparation of washed platelets, PRP was centrifuged at 640 *g* for 5 min at RT. The platelet pellet was resuspended in modified Tyrode-HEPES buffer (134 mM NaCl, 0.34 mM Na_2_HPO_4_, 2.9 mM KCl, 12 mM NaHCO_3_, 5 mM HEPES, 1 mM MgCl_2_, 5 mM glucose, and 0.35% bovine serum albumin [BSA; pH 7.4]) in the presence of prostacyclin (0.5 µM) and apyrase (0.02 U/mL). Platelets were finally resuspended in the same buffer without prostacyclin (pH 7.4; 0.02 U/mL apyrase) and incubated at 37 °C for 30 min before use^32^.

### Aggregometry

To determine platelet aggregation, light transmission was measured using washed platelets adjusted to a concentration of 0.5 × 10^6^ platelets/µL in the presence (U46619, collagen and rhodocytin) or absence (thrombin) of 70 µg/mL human fibrinogen (Sigma). PRP was used for ADP-induced aggregation. Light transmission was recorded over 10 min on an APACT (Hamburg, Germany).

### Transmission electron microscopy

Washed platelets were fixed with 2.5% glutaraldehyde in 50 mM cacodylate buffer (pH 7.2). After embedding in epon 812, ultra-thin sections were generated and stained with 2% uranyl acetate and lead citrate. Samples were analyzed on a Zeiss EM900 microscope. The visualization of the cytoskeleton of resting and spread platelets was performed as previously described^33^.

### Flow cytometry

For glycoprotein expression, heparinized whole blood was diluted in Tyrode’s-HEPES buffer and incubated with the respective fluorophore-conjugated antibodies. For activation studies, platelets in washed blood were activated with the indicated agonists and stained with the fluorophore-conjugated antibodies. To determine actin polymerization, washed platelets were incubated with a Dylight-649 labelled anti-GPIX antibody derivative (20 µg/mL) and either left unstimulated or were treated with thrombin for 2 min. Platelets were fixed with 10% PFA, permeabilized with 1% Triton X-100, stained with 10 µM phalloidin-fluorescein isothiocyanate for 30 min^7^. All analyses were performed on a FACSCalibur (BD Biosciences, Heidelberg, Germany).

### Immunofluorescence of resting platelets on Poly-L-lysine

Platelets were treated with 2% PFA and 0.1% IGEPAL CA-630. Phalloidin-Atto647N (0.075 pg/µL, Sigma-Aldrich) was used for F-actin staining. Alpha-tubulin was stained using Alexa546-conjugated anti-α-tubulin antibodies (200 µg/mL, Santa Cruz). MACF1 was detected using anti-MACF1^19^ (dilution: 1:400) and goat anti-rabbit IgG-Alexa 488 (2 mg/mL, Invitrogen) antibodies. Samples were visualized with a Leica TCS SP8 confocal microscope.

### Adhesion under flow conditions

Cover slips were coated with 200 µg/mL Horm collagen at 37 °C over night, washed with PBS and blocked with 1% BSA in PBS for 1 h at 37 °C. Blood was collected in heparin (20 U/mL) and further diluted (2:1) in Tyrode’s buffer supplemented with Ca^2+^, incubated with Dylight-488-conjugated anti-GPIX derivative (0.2 µg/mL) at 37 °C for 5 min. Transparent flow chambers with a slit depth of 50 µm, equipped with the coated cover slips, were connected to the blood filled syringe. Perfusion was performed at shear stress equivalent to a wall shear rate of 1,000 s^−1^. Blood was perfused for 4 min over the collagen coated surface and washed with Tyrode’s buffer for 4 min. Image analysis was performed using ImageJ software^34^.

### Platelet spreading

Coverslips were coated with 100 µg/mL human fibrinogen (Sigma-Aldrich). After addition of 0.01 U/mL thrombin (Roche), platelets were immediately allowed to spread on the coated surface. PFA-fixed platelets were imaged at a Zeiss Axiovert 200 microscope and analyzed using ImageJ software.

### Clot retraction

PRP (3 × 10^5^ platelets/µL) was incubated with bovine thrombin (Sigma-Aldrich 4 U/mL) and CaCl_2_ (20 mmoL/L) for 80 min.

### Immunoblotting

Platelet lysates were separated by SDS-PAGE and blotted onto polyvinylidene difluoride membranes. Membranes were incubated with an anti-MACF1 antibody (Bethyl Laboratories #A304-564A) over night. Horseradish peroxidase-conjugated secondary antibodies and enhanced chemiluminescence solution (MoBiTec) were used for visualization. Immunoblots were recorded directly using an Amersham Imager 600 (GE Healthcare) or with an Amersham Hyperfilm (GE Healthcare) and subsequent developing by a Cawomat 2000 IR apparatus (CAWO Solutions). Full length scanned immunoblots are shown in Suppl. Fig. 6.

### Bleeding time

A 2 mm segment of the tail tip of an anesthetized mouse was removed with a scalpel and blood drops were absorbed with a filter paper every 20 seconds^35^.

### Data analysis

Results are mean ± standard deviation. Differences between control and knockout mice were statistically analyzed using the Mann-Whitney-U test. *P*-values < 0.05 were considered as statistically significant: *P < 0.05; **P < 0.01. Results with a *P*-value > 0.05 were considered as not significant.

## Supplementary information

Supplementary Information

## Data Availability

The data generated and analyzed in this study are available from the corresponding author on reasonable request.
