# Supplementary material for: The cytoskeletal crosslinking protein MACF1 is dispensable for thrombus formation and hemostasis
Source: Sci Rep. 2019 May 22;9:7726. doi: 10.1038/s41598-019-44183-6 (PMC6531446; doi:10.1038/s41598-019-44183-6)
Supplement: Supplementary file 1 — Supplementary Information [file 41598_2019_44183_MOESM1_ESM.pdf]

SUPPLEMENTARY INFORMATION

The cytoskeletal crosslinking protein MACF1 is dispensable for thrombus formation and hemostasis

Short running title: MACF1 in platelet function

Yvonne Schurr, Markus Spindler, Hendrikje Kurz, Markus Bender*

Institute of Experimental Biomedicine – Chair I, University Hospital and Rudolf Virchow Center, Würzburg, Germany

*Correspondence to: Markus Bender, PhD; Institute of Experimental Biomedicine, Chair I, University Hospital Würzburg; Josef-Schneider-Str. 2, D15, 97080 Würzburg, Germany; Phone: +49 931-201-48328; E-mail: Bender_M1@ukw.de

SUPPLEMENTAL METHODS

Antibodies. One anti-MACF1 antibody was obtained from Elaine Fuchs (Rockefeller University; Supplemental Figure 1A left and middle)[1]. Another anti-MACF1 antibody was purchased from Santa Cruz (Supplemental Figure 1A right). Antibodies directed against the following proteins were purchased from the indicated companies: DAMM1 (Santa Cruz #sc-100942), mDia (Abcam #ab 129167), Gas2L1 (Invitrogen #PA521729), Wasp (CellSignaling #4860), ac-tubulin (Santa Cruz #sc-23950), alpha-tubulin (Sigma #T6074), Actin (Sigma #A2066), Rac1 (BD Biosciences #610650), Profililn1 (Sigma #P7749). Anti-GAPDH antibody (Sigma #G99545) served as loading control.

Histology. Five µm thick sections of formalin-fixed paraffin-embedded spleens were stained with hematoxylin and eosin (Sigma-Aldrich). Pictures of sections were recorded with an inverted Leica DMI 4000 B microscope.

Immunofluorescence. *Femora cryosections*: Femora were isolated, fixed with 4% PFA and 5 mM sucrose, transferred into 10% sucrose in PBS and dehydrated using a graded sucrose series. Subsequently, the samples were embedded in Cryo-Gel (Leica Biosystems) and frozen by -20°C. Seven µm thick cryosections were generated using the Kawamoto method[2] and probed with Alexa488-conjugated anti-GPIX antibodies (1.33 µg/mL Xia.B4, Emfret Analytics) to label platelets and megakaryocytes (MKs), and Alexa647-conjugated anti-CD105 antibodies (3.33 mg/mL MJ7/18, Biolegend) to stain the endothelium. Nuclei were stained using Fluoroshield with DAPI (Sigma-Aldrich). Samples were visualized with a Leica TCS SP5 confocal microscope. *Platelets:* Washed platelets were fixed and permeabilized with 2% PFA and 0.1% IGEPAL CA-630 in PBS. F-actin was stained using phalloidin-Atto647N (0.075 pg/µL, Sigma-Aldrich). Alpha-tubulin was stained using Alexa488-conjugated anti-α-tubulin antibodies (1 µg/mL, Thermo Fisher). For cold-induced microtubule disassembly, washed platelets ($3x10^5$ platelets/µL) were incubated at 4°C for 3 h to induce microtubule depolymerization. Microtubule reassembly was allowed by rewarming at 37°C for 30 min before subsequent fixation and staining. Samples were visualized with a Leica TCS SP5 confocal microscope.

In vitro differentiation and cultivation of fetal-liver derived MKs. Fetal liver cells of embryos at day 13.5 to 14.5 were cultured in medium (Iscove Modified Dulbecco medium, 10% fetal calf serum, and 1% penicillin/streptomycin) containing thrombopoietin (TPO) from cell culture supernatant. On day 3, MKs were enriched by gradient density filtration with 1.5% and 3% bovine serum albumin. Day 4 MKs were analyzed and counted for proplatelet formation under a light microscope.

Determination of MK ploidy. Bone marrow was harvested and MKs were stained with the MK-specific antibody (anti-GPIb) and DNA with propidium iodide. DNA distribution was determined by flow cytometric analyses on a FACSCalibur.

SUPPLEMENTAL FIGURE LEGENDS

Supplemental Figure 1

A

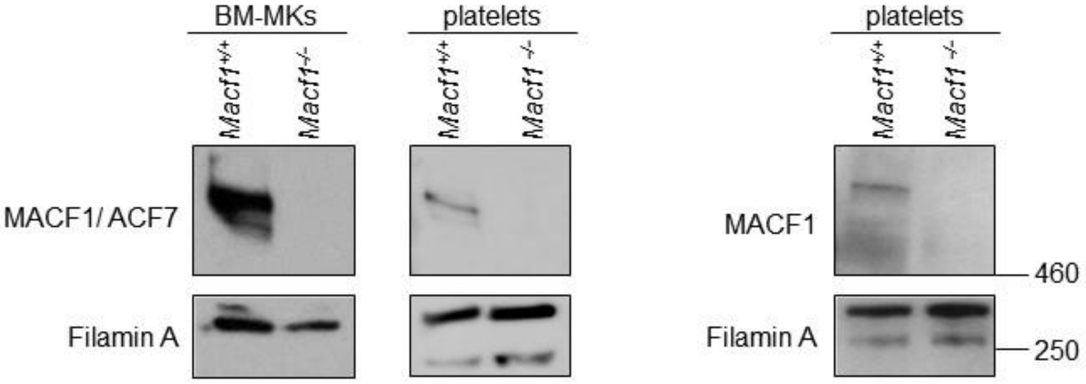

B

Parameter	*Macf1*$^{+/+}$	*Macf1*$^{-/-}$	*p*
Platelets (10^3/µL)	867.80 ± 57.15	826.33 ± 23.7	0.10
MPV (fL)	4.88 ± 0.15	5.00 ± 0.22	0.71
Leukocytes (10^3/µL)	6.66 ± 1.25	4.67 ± 1.91	0.16
Neutrophils (10^3/µL)	2.45 ± 1.23	2.26 ± 1.52	0.74
Lymphocytes (10^3/µL)	3.92 ± 1.23	2.17 ± 1.44	0.13
Monocytes (10^3/µL)	0.28 ± 0.09	0.21 ± 0.23	0.99
Eosinophils (10^3/µL)	0.02 ± 0.01	0.02 ± 0.02	0.86
Erythrocytes (10^3/µL)	9.55 ± 0.63	10.01 ± 0.47	0.82
MCV (fL)	45.42 ± 1.40	44.40 ± 0.33	0.79

Supplemental Figure 1 │ MACF1 deficiency in MKs and platelets has no effect on whole blood parameters

(A) MACF1 expression in MKs and platelets was assessed by Western blot analysis. Anti-MACF1 antibodies: (left, middle) Antibody was kindly provided by Elaine Fuchs (Rockefeller University), (right) Antibody was purchased from Santa Cruz (#sc-377532). Filamin A (Cell Signalling #cs-4762) served as loading control. (n = 2-3) **(B)** Whole blood parameters determined by a hematology analyser. (n = 3, two independent experiments)

Supplemental Figure 2

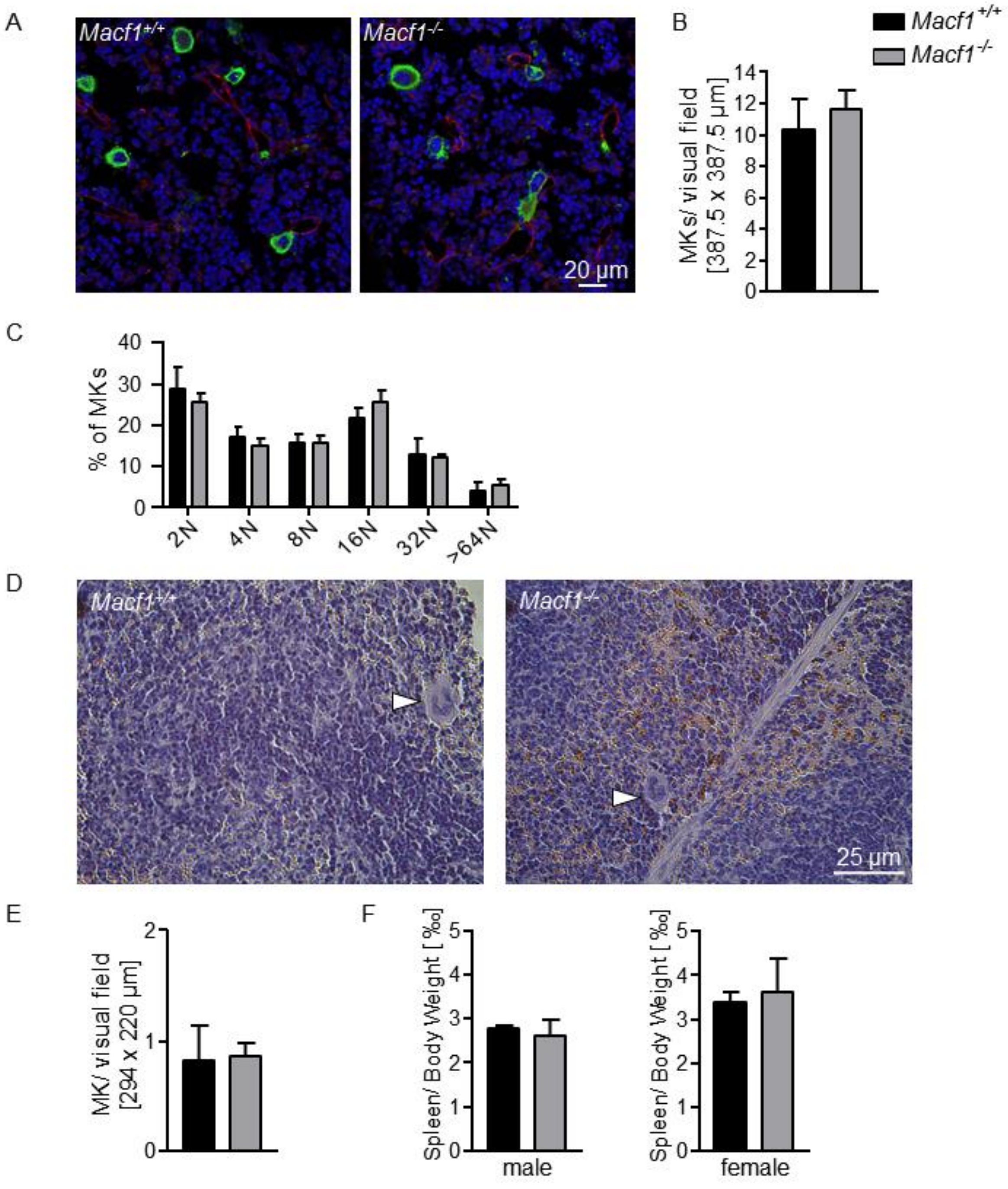

Supplemental Figure 2 │Normal MK appearance in the bone marrow and spleen of MACF1 deficient mice

Representative bone marrow cryosections are shown in **(A)**; blue: DAPI, red: CD105/vessels, green: GPIX/MKs. (n = 3). MKs were quantified in the bone marrow per visual field **(B)**. **(C)** Bone marrow MKs were stained with a MK-specific antibody (anti-GPIb) and DNA with propidium iodide. Ploidy was assessed by flow cytometry. (n = 3) **(D)** H&E staining of the

spleen is shown. Arrowheads indicate MKs. (n = 4) **(E)** MKs were quantified per visual field. **(F)** Determination of spleen to body weight in 9 week old male and 12 week old female mice. (n = 4)

Supplemental Figure 3

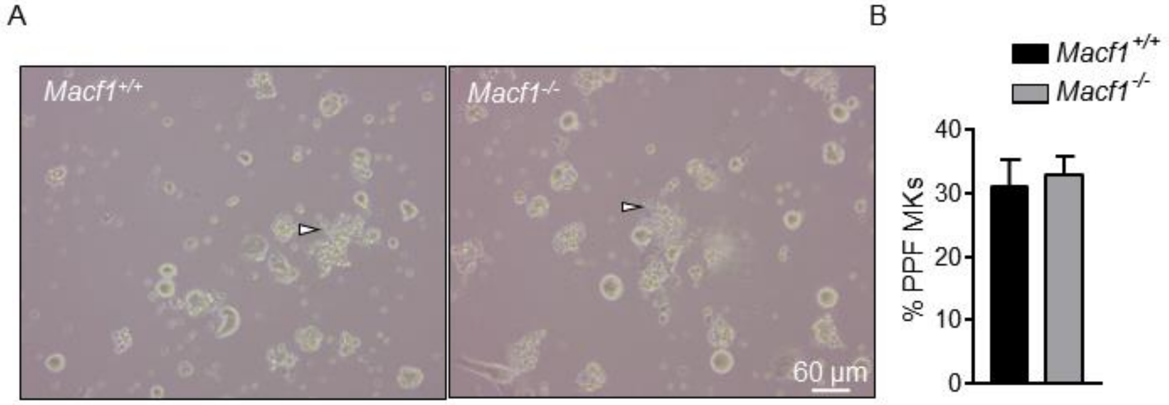

Supplemental Figure 3 │Normal proplatelet formation of MACF1 deficient MKs in vitro

(A) Representative images of cultured fetal liver cells are shown. Arrowheads indicate proplatelets. **(B)** Percentage of proplatelet forming cells (PPF) was quantified per visual field. (n = 3)

Supplemental Figure 4

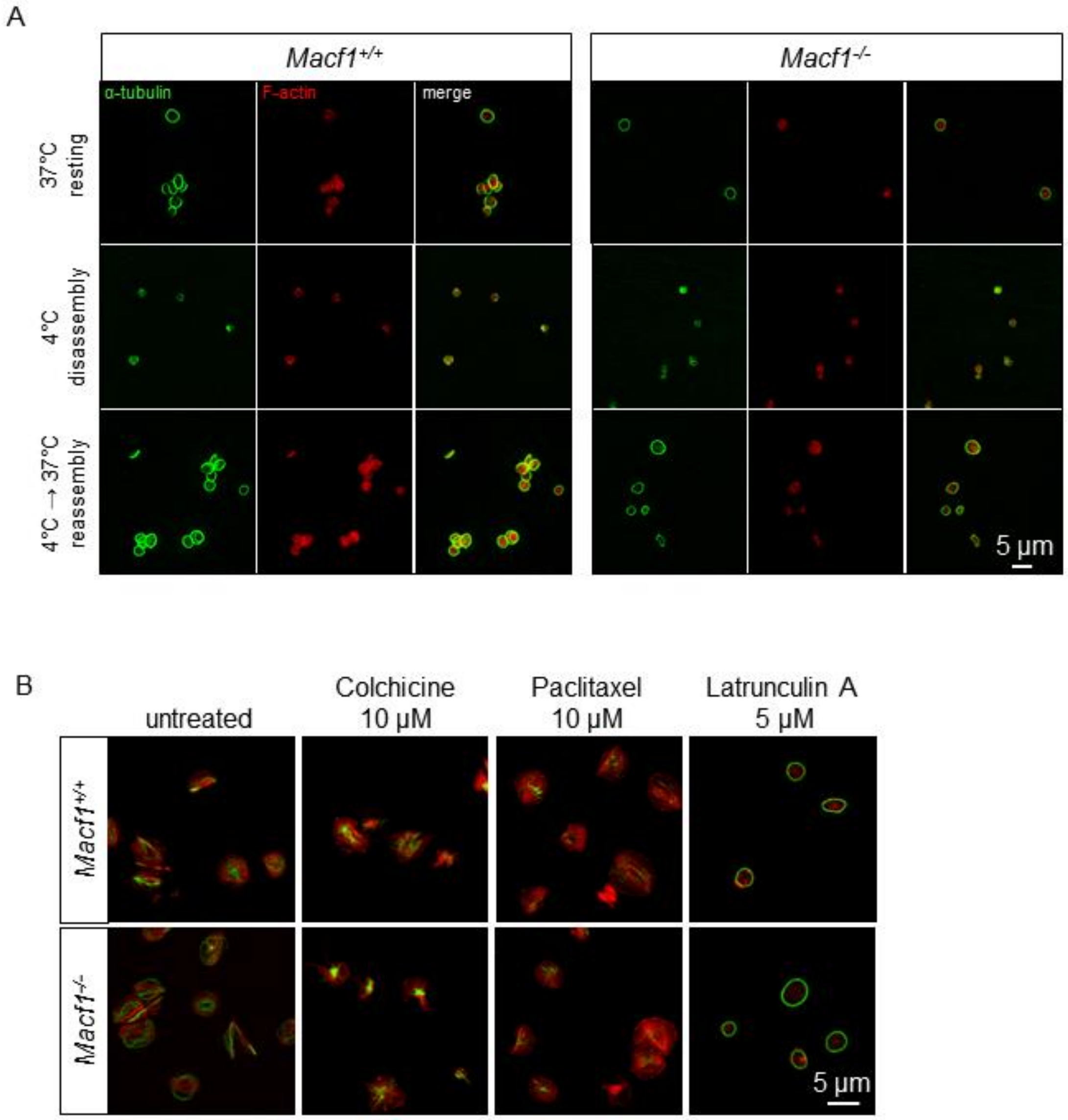

Supplemental Figure 4│Lack of MACF1 does not affect microtubule and actin dynamics

(A) *Macf1*$^{+/+}$ and *Macf1*$^{-/-}$ platelets were incubated for 3 h at different temperatures to induce dis- or reassembly of microtubules, and stained for F-actin and α-tubulin. **(B)** Platelets pre-treated with indicated cytoskeletal-modifying toxins were allowed to spread on fibrinogen for 30 min. Green: α-tubulin, red: F-actin. (n = 3, representative for three independent experiments)

Supplemental Figure 5

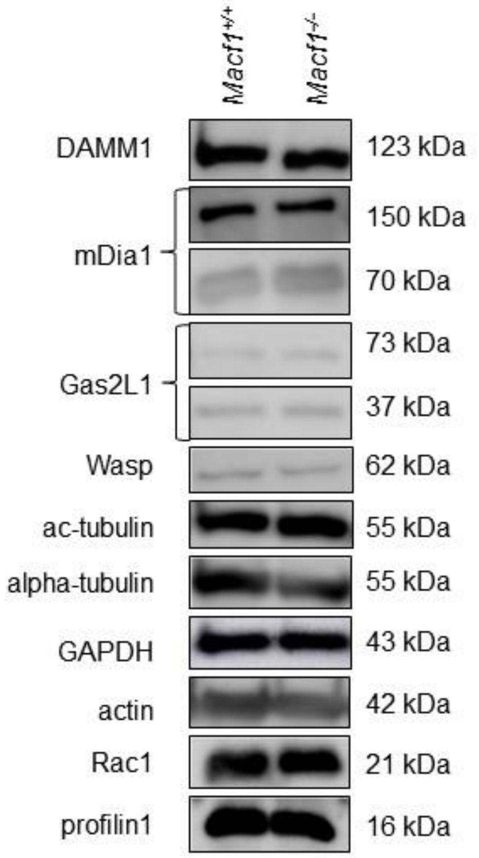

Supplemental Figure 5 │Normal expression of other cytoskeletal regulating proteins in MACF1 deficient platelets

Expression of different cytoskeleton regulatory proteins in *Macf1*$^{+/+}$ and *Macf1*$^{-/-}$ platelets was assessed by Western blot analysis. GAPDH served as loading control. (n = 3)

Supplemental Figure 6

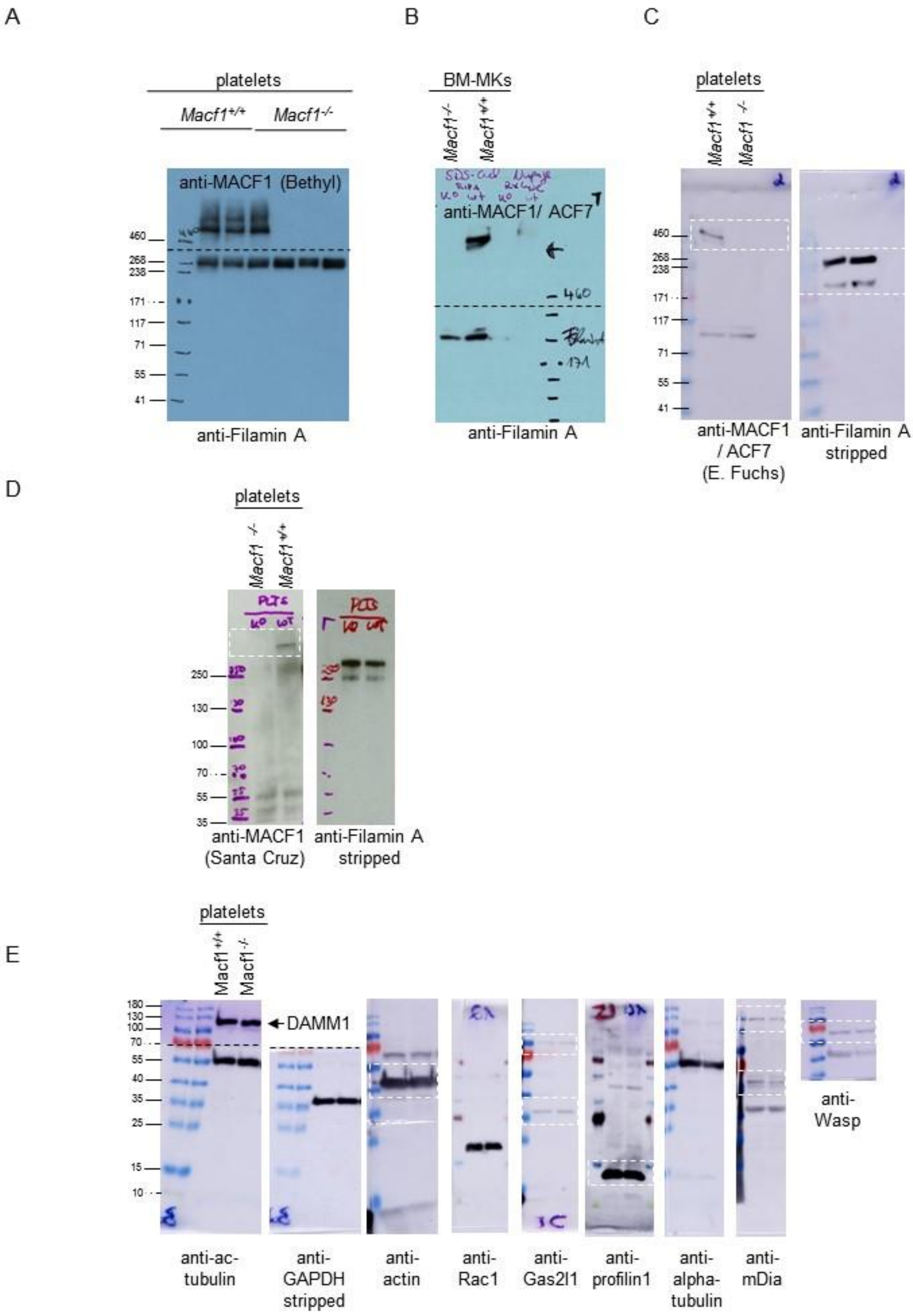

Supplemental Figure 6 │Full length immunoblots

Immunoblots were recorded with an Amersham Hyperfilm (GE Healthcare) and a Cawomat 2000 IR developing apparatus (A, B, D) or directly using an Amersham Imager 600 (GE Healthcare) (C, E). Black dashed lines indicate that membrane was cut. White dashed boxes indicate bands at expected protein size. Different protein ladders were used as indicated.

References

1. Karakesisoglou, I., Yang, Y. & Fuchs, E. An epidermal plakin that integrates actin and microtubule networks at cellular junctions. *J. Cell Biol.* **149,** 195–208 (2000).

2. Kawamoto, T. Use of a new adhesive film for the preparation of multi-purpose fresh-frozen sections from hard tissues, whole-animals, insects and plants. *Arch. Histol. Cytol.* **66,** 123–143 (2003).
